# Integrated Phenotypic and Genome‐Informed Characterization of Environmental *Aspergillus* Isolates With Potential for Citric Acid Production

**DOI:** 10.1002/mbo3.70452

**Published:** 2026-11-05

**Authors:** Wimbélé Epiphanie Kouman, Okran Beyosse Christophe Kacou, Sansan Kambou, Ousmane Fofana, Djédoux Maxime Angaman

**Affiliations:** ^1^ Department of Biochemistry‐Microbiology Jean LOROUGNON GUEDE University Daloa Côte d'Ivoire

**Keywords:** Aspergillus niger species complex, citric acid biosynthesis, environmental fungal isolates, fungal genomics, illumina sequencing, industrial biotechnology

## Abstract

*Aspergillus* species are widely used in industrial biotechnology, particularly for citric acid production. This study characterized environmental *Aspergillus* isolates from Côte d'Ivoire with potential for citric acid production using an integrated phenotypic and genome‐informed approach. Isolates recovered from banana rhizosphere soil and decomposing fruits and vegetables were screened for aflatoxin‐associated reactions using the ammonia vapor assay and for acidification on bromocresol green‐supplemented Czapek‐Dox agar. Selected isolates were analyzed by whole‐genome sequencing, Kaiju taxonomic profiling, and EggNOG‐mapper functional annotation. Citric acid production was quantified under solid‐state fermentation using a calibrated colorimetric assay. Among 110 fungal isolates recovered, 24 *Aspergillus* isolates were grouped into seven morphotypes. Four isolates (E1‐E4) showed acidification activity and no detectable aflatoxin‐associated reaction under the assay conditions. Morphological and whole‐genome taxonomic analyses conservatively affiliated E1‐E3 with the *Aspergillus niger* species complex, whereas E4 remained taxonomically unresolved. E2 showed the highest proportion of reads assigned to *A. niger* reference sequences (92%) and the greatest representation of predicted functions associated with citrate metabolism and carbohydrate degradation. During solid‐state fermentation, E2 exhibited the highest measured citric acid concentration under the tested conditions. Comparative genomic analyses also showed greater similarity among E1‐E3 than with E4. Overall, E2 emerged as the most promising candidate for further citric acid production studies, supporting the value of combining quantitative phenotypic assessment with genome‐informed characterization of environmental fungal isolates.

## Graphical abstract

Environmental *Aspergillus* isolates recovered from banana rhizosphere soil and decomposing fruits and vegetables in Côte d'Ivoire were evaluated using an integrated phenotypic and genome‐informed approach. Four isolates (E1‐E4) showing acidification capacity and no detectable aflatoxin‐associated reaction were selected for further characterization. Morphological and whole‐genome taxonomic analyses conservatively affiliated E1‐E3 with the *Aspergillus niger* species complex, whereas E4 remained taxonomically unresolved. Functional genomic analyses revealed predicted functions associated with citrate metabolism and carbohydrate degradation, with E2 showing the strongest genomic potential. Quantitative solid‐state fermentation further showed the highest citric acid production for E2 under the tested conditions. Overall, E2 emerged as the most promising candidate for further citric acid production studies and subsequent bioprocess optimization.

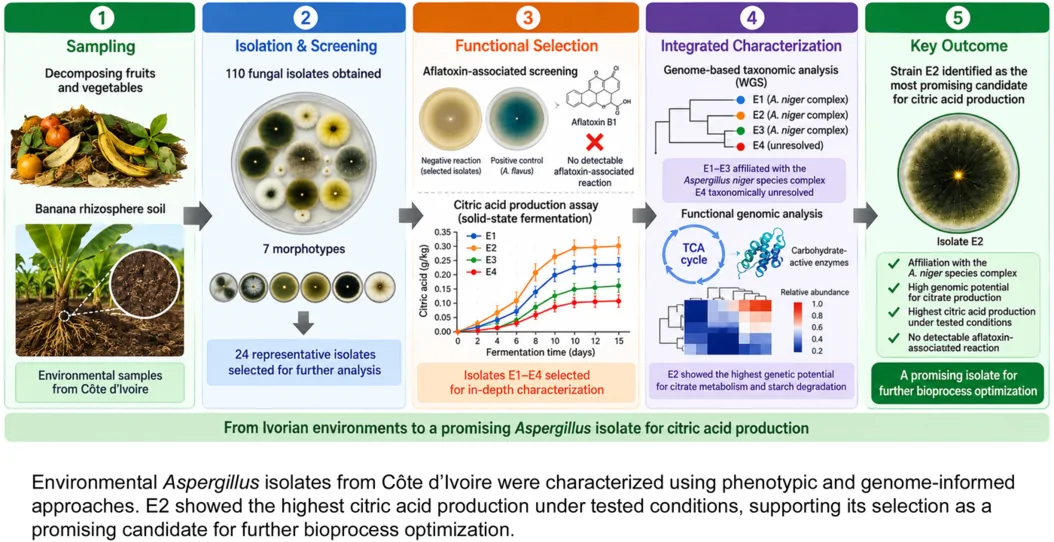

## 1 Introduction

Filamentous fungi are among the most important microorganisms used in industrial biotechnology because of their ability to produce a wide range of valuable metabolites, including organic acids, enzymes, antibiotics, and bioactive compounds (Chroumpi et al. 2020; Füting et al. 2021). Their metabolic versatility, efficient secretion systems, and capacity to grow on inexpensive substrates have made them essential microbial cell factories for numerous industrial applications (Tiwari and Dufossé 2023).

Among filamentous fungi, species belonging to the genus *Aspergillus* have attracted considerable attention due to their economic importance and well‐characterized metabolic pathways (Samson et al. 2014). In particular, *Aspergillus niger* is recognized as one of the most efficient microbial producers of citric acid, a compound extensively used in the food, pharmaceutical, cosmetic, and chemical industries (Behera 2020; Tong et al. 2019). Global citric acid production exceeds two million tons annually, with *A. niger* accounting for the vast majority of industrial production because of its high yield, genetic stability, and ability to utilize a wide variety of carbon sources (Latif et al. 2025).

The continuous search for new *Aspergillus* strains remains an important objective in industrial microbiology. Environmental isolates may harbor unique physiological and metabolic characteristics that can improve substrate utilization, enzyme production, or organic acid biosynthesis (Silva et al. 2020). Agricultural soils, rhizosphere environments, and decomposing plant materials constitute particularly rich ecological niches for *Aspergillus* species because they provide abundant carbon sources and selective pressures that shape fungal adaptation (Pitt and Hocking 2009; E. et al. 2002). Consequently, environmental screening remains a valuable strategy for identifying strains with potential biotechnological applications.

However, the exploitation of environmental *Aspergillus* isolates presents several challenges. First, species belonging to the *Aspergillus* genus exhibit substantial phenotypic and genetic diversity, particularly within the *Aspergillus niger* species complex, making accurate taxonomic identification difficult (Samson et al. 2014; Silva et al. 2020). Second, some *Aspergillus* species are capable of producing mycotoxins that may compromise their safe use in industrial processes (Bennett and Klich 2003; Frisvad et al. 2019). Therefore, the selection of candidate strains requires not only the evaluation of their metabolic potential but also careful consideration of their taxonomic identity and biosafety characteristics.

Traditionally, the selection of *Aspergillus* isolates has relied on morphological observations and biochemical screening methods, including tests for acid production and mycotoxin detection (Balajee et al. 2007; Diguta 2010). Although these approaches remain useful for preliminary characterization, they provide limited information regarding the genetic basis of metabolic capabilities and may not accurately reflect taxonomic relationships among closely related species (Nilsson et al. 2019). Recent advances in genome sequencing technologies and bioinformatic tools have significantly improved fungal characterization by enabling detailed taxonomic profiling and functional annotation of genes involved in central metabolism, carbohydrate degradation, and organic acid biosynthesis (Cairns et al. 2019; Galagan et al. 2005).

Integrating conventional microbiological screening with genome‐informed analyses may therefore provide a more comprehensive framework for the preliminary selection of fungal isolates of biotechnological interest. Such an approach allows phenotypic observations to be complemented by taxonomic and functional information, thereby improving the identification of promising candidate strains for future industrial evaluation.

In this context, the present study aimed to isolate *Aspergillus* strains from environmental samples collected in Côte d'Ivoire and to characterize isolates showing potential for citric acid production using an integrated phenotypic and bioinformatic approach. Selected isolates were evaluated for acidification capacity, screened for detectable aflatoxin‐associated reactions, and further investigated through whole‐genome sequencing, taxonomic profiling, and functional annotation to assess their genetic consistency and metabolic potential.

## 2 Materials and Methods

### 2.1 Sample Collection

Environmental samples were collected from two ecological niches known to harbor filamentous fungi: decomposing fruits and vegetables and banana rhizosphere soils. Fruit and vegetable samples consisted of lemon (*Citrus limon*), onion (*Allium cepa*), and carrot (*Daucus carota*) collected near market‐waste disposal areas in Yopougon (5.33926° N, 4.076° W), Koumassi (5°18′12″ N, 4°00′33″ W), and Grand‐Bassam (5°12′00″ N, 3°44′00″ W), Côte d'Ivoire. For each fruit or vegetable type, 10 independent samples were collected at each of the three sampling sites, resulting in 30 onion, 30 lemon, and 30 carrot samples. In addition, 10 independent banana rhizosphere soil samples were collected from banana plantations in Bonoua (5°28′ N, 3°12′ W), Côte d'Ivoire. Thus, a total of 100 independent environmental samples were analyzed.

Soil sampling was conducted during the rainy season, and the soil temperature at the time of collection was approximately 25°C. All samples were collected aseptically into sterile containers, transported to the laboratory under refrigerated conditions, and processed within 24 h of collection.

### 2.2 Isolation of Filamentous Fungi

Fungal isolation was performed according to the standard method described by Iqbal et al. (2015). For soil samples, 10 g of soil were suspended in 90 mL of buffered peptone water to prepare the initial suspension. Serial decimal dilutions were then prepared by transferring 1 mL of the previous suspension into 9 mL of sterile distilled water until the desired dilution was obtained. One milliliter of the 10^−3^ and 10^−4^ dilutions was inoculated into sterile Petri dishes, followed by the addition of 20 mL of Potato Dextrose Agar (PDA) medium. The plates were gently homogenized and allowed to solidify. For the fruits and vegetables, fungal isolation was carried out using an air bio‐collector (PACK TRIO BAS MONO HEPA‐100 L/min). Each Petri dish containing Potato Dextrose Agar (PDA) medium was exposed sequentially to the air bio‐collector. Air from containers holding the decomposing samples was drawn at a flow rate of 100 L/min and evenly deposited onto the agar surface. Approximately six Petri dishes (90 mm diameter), each containing 20 mL of PDA medium, were used per sample. All plates were incubated at 30°C for 3–5 days.

### 2.3 Purification and Preservation of Strains

Presumptive filamentous fungal isolates were sub‐cultured and purified on Sabouraud agar supplemented with chloramphenicol. Purification was performed by successive streaking of individual colonies onto fresh agar plates to obtain axenic cultures. Plates were incubated at 30°C for 24 h. Pure cultures were confirmed based on macroscopic colony morphology and uniform growth characteristics. Purified isolates were then maintained on Czapek agar slants and stored for further analyses. A total of 110 filamentous fungal isolates were recovered, among which 24 were assigned to the genus *Aspergillus* and retained for further characterization.

### 2.4 Macroscopic and Microscopic Identification of Fungi

The isolates obtained were subcultured again onto Chloramphenicol Sabouraud agar. Macroscopic identification was performed visually and was based primarily on cultural characteristics. After culturing the isolates for 24 h at 37°C on Chloramphenicol Sabouraud agar, several aspects of the vegetative apparatus were observed. Furthermore Microscopic identification of *Aspergillus* sp. colonies was carried out based on their morphological characteristics and lactophenol blue staining of cotton according to standard methods of Diguta (2010).

### 2.5 Fungal Inoculum Preparation

The selected *Aspergillus* isolates were grown on Potato Dextrose Agar (PDA) plates (90 mm in diameter) and incubated at 30°C for 5 days until profuse sporulation occurred. Conidia were harvested from the mycelial surface using sterile distilled water and a sterile scraper. The resulting spore suspension was filtered to remove mycelial debris, and conidia were quantified using a Malassez hemocytometer. The suspension was then standardized by dilution with sterile distilled water to achieve a final concentration of approximately 1 × 10^7^ spores/mL.

### 2.6 Screening for Aflatoxin‐Associated Reactions

The ability of the isolated fungi to produce aflatoxin was detected in the laboratory using the method of Saito and Machida (1999). For this assay, isolates were inoculated onto Coconut Extract Agar, prepared by boiling 100 g of grated coconut in 1 L of distilled water for 15 min, filtering the liquid, and adding 20.0 g of agar prior to sterilization. Each strain was inoculated into the center of the solidified agar medium in glass Petri dishes and incubated at 30°C for 3 days. To observe the color change, the dishes were inverted, and 0.2 mL of a 25% ammonia solution was placed in the lid. The dishes were incubated for an additional 3 days at 30°C. Color shifts on the colony reverse were recorded across replicates using a standardized qualitative scale categorized as no detectable reaction (ND) or positive coloration matching a very strong reaction (++++).

### 2.7 Qualitative Screening of Acidification Capacity

A qualitative screening of citric acid production by the *Aspergillus* isolates was performed according to the standard method of Iqbal et al. (2015), with modifications described by Wang et al. (2020). The modified Czapek‐Dox agar medium was prepared with the following composition per liter of distilled water: 30.0 g sucrose, 2.0 g NaNO_3_, 1.0 g K_2_HPO_4_, 0.5 g MgSO_4_·7H_2_O, 0.5 g KCl, 0.01 g FeSO_4_, and 20.0 g agar. The initial pH of the medium was adjusted to 6.0. Following sterilization, the medium was cooled to 50°C–55°C. A separate indicator solution, prepared by dissolving 0.1 g of bromocresol green in 40 mL of sterile distilled water, was aseptically added to the liquid medium and mixed thoroughly to ensure an even distribution (final indicator concentration of 0.1 g/L). The medium was then poured into individual sterile Petri dishes in triplicate. After solidification, 0.5 mL of the standardized *Aspergillus* conidial suspension was inoculated onto each dish. The plates were incubated at 30 ± 1°C for 3 to 5 days. Fungal colonies producing yellow halo areas on the dishes were considered positive for citric acid production.

### 2.8 Quantitative Citric Acid Production

Solid‐state fermentation was conducted using industrial pineapple waste (DIAaa) as the solid substrate. The citric acid concentration in the extracts was quantified using the colorimetric method of Marier and Boulet (1958), as adapted for *Aspergillus* cultures by Dhillon et al. (2012). Briefly, 2.5 g of the fermented substrate was sampled at specific time points (Days 0, 2, 4, 6, 8, 10, 12, and 15) and dissolved in 12.5 mL of distilled water. The mixture was homogenized using a vortex mixer and centrifuged at 3000 rpm for 10 min. A 1.0 mL aliquot of the resulting extract was reacted with 1.3 mL of pyridine and 5.7 mL of acetic anhydride, and the absorbance was measured using a UV spectrophotometer at a wavelength of 420 nm. Quantitative values were calculated using a commercial citric acid standard curve (*y* = 0.186x + 0.0442, (*R* ^2^ = 0.997), where y represents the absorbance and x represents the concentration (*C*_*lue*_) in g/L. The final concentration was calculated in grams per kilogram of fermented substrate (g/kg) according to the relation:

$$C=\left(C_{lue}V\right)/m$$

*C*: the real concentration of citric acid in the substrate (g/kg), *C*_*lue*_: the concentration read from the spectrophotometer calibration curve (g/L), *V*: the extraction recovery volume (L), *m*: the initial mass of the fermented solid substrate sample (kg).

### 2.9 Genomic DNA Extraction

Genomic DNA extraction was performed according to a modified CTAB protocol described by Huang et al. (2018). The mycelium from 3‐day fungal cultures was ground and then degraded with CTAB1 buffer at 60°C in the presence of RNase and proteinase K to remove contaminants. After centrifugation, the nucleic acids were purified by a series of steps including chloroform extraction, selective precipitation with CTAB2, followed by a further chloroform extraction. The DNA was then precipitated with cold isopropanol, washed with 70% ethanol, dried, and finally suspended in a storage buffer before being stored at −20°C.

### 2.10 Whole‐Genome Sequencing

High molecular weight genomic DNA was extracted, and its quality and integrity were then verified by NanoDrop spectrophotometry, Qubit fluorimetry (ThermoFisher Scientific Q33238) with the Qubit 1X dsDNA HS Assay kit (ThermoFisher Scientific Q33230) and agarose gel electrophoresis to confirm DNA fragments larger than 20 kb. Sequencing libraries were prepared using the NEBNext Ultra II FS DNA Library Prep Kit for Illumina (New England Biolabs, Ipswich, MA, USA), according to the manufacturer's instructions. Library quality and fragment size were assessed by capillary electrophoresis and Qubit fluorimetry. Sequencing was performed on an Illumina NextSeq platform using a 300‐cycle sequencing configuration, generating 2 × 150‐bp paired‐end reads.

### 2.11 Bioinformatic Analyses

Raw data quality control was performed using FastQC, followed by the removal of low‐quality sequences and residual adapters using Trim Galore (Andrews 2010). Genome assembly was performed using SPAdes, and assembly quality was assessed using QUAST (Bankevich et al. 2012). Taxonomic profiling was performed using Kaiju against the RefSeq Fungi database.

Functional annotation was performed using EggNOG‐mapper against the eggNOG orthology database. Predicted protein functions were subsequently cross‐referenced with the KEGG Citrate Cycle/Tricarboxylic Acid Cycle pathway (map00020) to identify annotations associated with citrate metabolism. Particular attention was given to annotations corresponding to citrate synthase (EC 2.3.3.1), aconitase (EC 4.2.1.3), isocitrate dehydrogenase (EC 1.1.1.41/42), and isocitrate lyase (EC 4.1.3.1) (Cantalapiedra et al. 2021).

### 2.12 Comparative Genomic Analyses

Comparative analyses among isolates E1‐E4 were conducted using taxonomic assignments, predicted functional profiles, and shared genomic features. Similarity relationships among isolates were visualized using Venn diagrams and hierarchical clustering based on genomic distance matrices derived from annotated gene content.

These analyses were used to explore genomic relatedness and similarities in predicted functional profiles among the candidate isolates. Because these comparisons were based on genome‐derived annotations, they were interpreted as descriptive genomic relationships rather than as definitive phylogenetic evidence.

### 2.13 Statistical Analysis

All experimental assays were performed independently in triplicate (*n* = 3), and quantitative data were expressed as mean values ± standard deviation. To evaluate significant differences among the selected fungal isolates, quantitative datasets—specifically the yellow acidification halo diameters and the 15‐day solid‐state fermentation kinetics—were subjected to one‐way Analysis of Variance (ANOVA). Mean separations were determined using Tukey's honestly significant difference (HSD) post‐hoc test. All statistical operations, data modeling, and graphical layouts were conducted utilizing the R software framework (v4.2.2) integrated within the RStudio integrated development environment (IDE), deploying core statistical computational packages. Statistical significance for all parametric tests was strictly established at a threshold baseline of (*α* = 0.05).

## 3 Results and Discussion

### 3.1 Diversity and Morphological Characterization of Fungal Isolates

A total of 110 filamentous fungal isolates were recovered from environmental samples, including 70 isolates from decomposing fruits and vegetables and 40 isolates from banana rhizosphere soils (Table 1). Among these isolates, 24 were preliminarily assigned to the genus *Aspergillus* based on their macroscopic and microscopic characteristics and were subsequently grouped into seven morphotypes (Figure 1).

**Table 1:** Distribution of fungal isolates and Aspergillus spp. recovered from different environmental sample sources in Côte d'Ivoire.

Sample source	Biological material	Total fungal isolates	*Aspergillus* isolates
Rhizosphere soil	Banana rhizosphere	40	4
Fruits and vegetables	Onion	30	9
	Lemon	15	5
	Carrot	25	6
Total	—	110	24

*Note:* Environmental samples included banana rhizosphere soils and decomposing vegetables (onion, lemon, and carrot). Values represent the number of isolates recovered after culture and purification.

**Figure 1:**
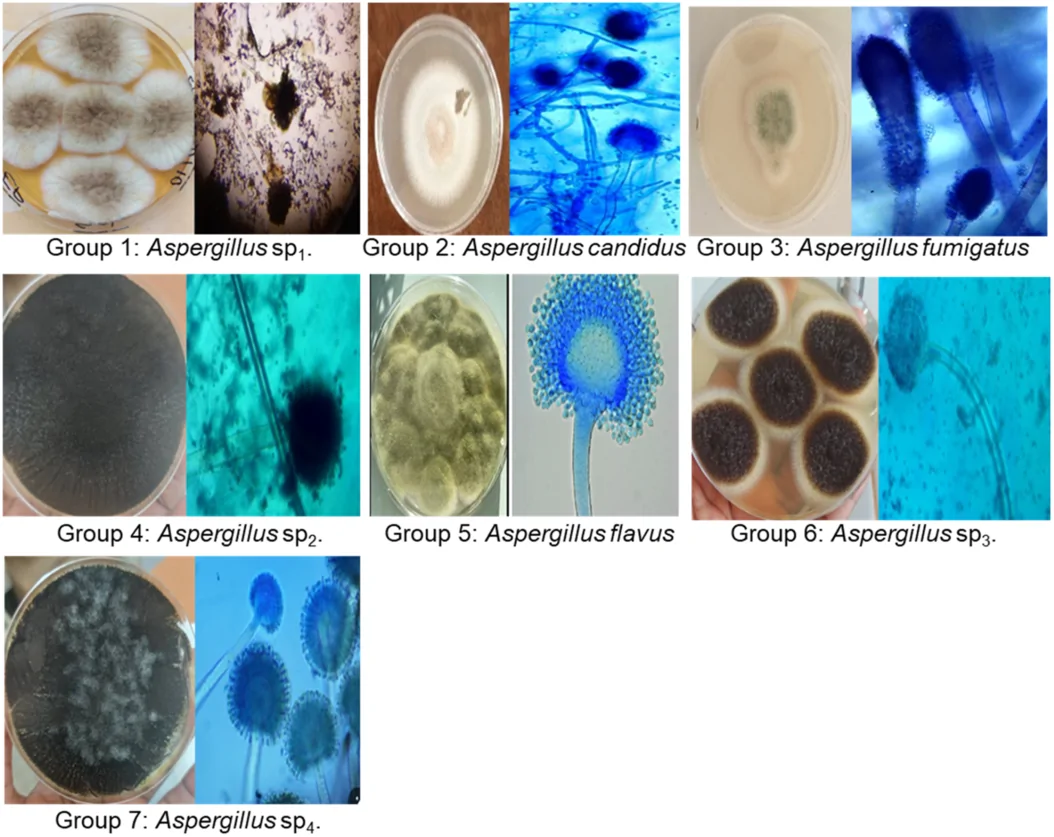
Macroscopic and microscopic characteristics of *Aspergillus* morphotypes isolated from environmental samples after 5 days of incubation. Representative colony morphology (macroscopic view) and microscopic structures of the seven *Aspergillus* morphotypes recovered from banana rhizosphere soils and decomposing fruits and vegetables. Cultures were incubated for 5 days on PDA medium, and microscopic observations were performed after lactophenol cotton blue staining.

The predominance of fungal isolates in decomposing fruits and vegetables may be explained by the high availability of simple carbohydrates and favorable moisture conditions that promote fungal growth. Similar observations have been reported in studies describing decomposing plant materials as important reservoirs of filamentous fungi, particularly species belonging to the genera *Aspergillus*, *Penicillium*, and *Rhizopus* (Pitt and Hocking 2009).

The seven morphotypes exhibited differences in colony coloration, texture, sporulation patterns, and conidial morphology, highlighting the phenotypic diversity of environmental *Aspergillus* populations. Such variability is commonly observed within the *Aspergillus niger* species complex and reflects adaptation to different ecological niches (Samson et al. 2014).

To strengthen the morphological characterization, detailed microscopic measurements and morphometric profiles were recorded for the seven identified environmental morphotypes, which include the specific target isolates selected for subsequent genomic analysis (Table 2). Microscopic evaluations were performed based on 10 randomly selected conidial structures per isolate to establish representative size intervals. Among the selected strains, isolate E1 (*Aspergillus* sp. 1) was characterized by a vesicle diameter of 35.0–40.0 µm, a biseriate phialide arrangement, and smooth mature conidia (2.8–3.5 µm). Isolate E2 (*Aspergillus* sp. 2) exhibited a typical *Aspergillus niger* complex phenotype, presenting larger vesicles (40.0–55.0 µm), a biseriate arrangement, and very rough conidia (3.5–4.5 µm). A similar biseriate structure and radiate head configuration were recorded for isolate E3 (*Aspergillus* sp. 3). In contrast, the divergent isolate E4 (*Aspergillus* sp. 4) was clearly distinguished by a uniseriate phialide arrangement, a smaller vesicle diameter (20.0–35.0 µm), and a loose columnar head structure.

**Table 2:** Morphometric and microscopic characteristics of the Aspergillus morphotypes and target isolates.

Morphotype/Isolate	Vesicle diameter (µm)	Phialide arrangement	Conidia texture	Conidia size (µm)	*Aspergillus* head
*Aspergillus flavus* (O5)	20–35	Uniseriate and biseriate	spiny to rough	3,5 ‐ 5,0	Radiate to loose
*Aspergillus fumigatus*	20–30	Uniseriate	Finely spiny	2,5 ‐ 3,5	Columnar
*Aspergillus candidus* (A2)	30–40	Biseriate	Smooth walls	2,5 ‐ 3,5	Globular, radiate
*Aspergillus* sp. 1 (Isolate E1)	35–40	Biseriate	Smooth	2,8 ‐ 3,5	Radiate
*Aspergillus* sp. 2 (Isolate E2)	40–55	Biseriate	Very rough	3,5 ‐ 4,5	Detached, radiate
*Aspergillus* sp. 3 (Isolate E3)	35–40	Biseriate	Spiny	3,2 ‐ 4,0	Radiate
*Aspergillus* sp. 4 (Isolate E4)	20–35	Uniseriate	Rough	3,0 ‐ 3,8	Loose column

*Note:* Microscopic and diagnostic morphological characteristics were recorded for the seven identified environmental *Aspergillus* morphotypes. For each morphotype and target isolate (E1–E4), data represent the specific intervals for vesicle dimensions, phialide seriation arrangements (uniseriate or biseriate), conidial surface textures, mature conidial sizes, and overall structural configurations of the *Aspergillus* heads. On day 5 of incubation on PDA at 30°C, macroscopic colony diameters ranged from 60 to 78 mm for isolates E1–E3, and 40 to 45 mm for isolate E4. To ensure statistical accuracy, all microscopic measurements were performed on 10 randomly selected conidial structures per isolate using standard mycological ocular micrometers.

From a taxonomic perspective, the biseriate phialide arrangement, radiate head configurations, and rough‐walled conidia recorded for E1, E2, and E3 represent standard morphological markers associated with the *Aspergillus niger* species complex (Section *Nigri*). These morphological characteristics are consistent with descriptions of black aspergilli belonging to *Aspergillus* section *Nigri* (Samson et al. 2014). Conversely, the strictly uniseriate phialide arrangement and smaller vesicle architecture of isolate E4 indicate a distinct morphological profile compared with E1‐E3. However, these morphological differences alone are insufficient to support assignment of E4 to a specific *Aspergillus* section or species. In the context of this study, these structural variations provided a phenotypic basis for selecting E4 for further molecular and whole‐genome characterization within a polyphasic taxonomic framework (Hamzehee et al. 2025).

### 3.2 Screening for Aflatoxin‐Associated Reactions and Acidification Capacity

The standardized ammonia vapor assay performed on Coconut Extract Agar (CEA) revealed clear differences among the isolates regarding detectable aflatoxin‐associated reactions. Isolates A2 (*A. candidus*) and O5 (*A. flavus*) exhibited very strong positive reactions (++++), whereas isolates E1, E2, E3, E4, and G3 showed no detectable reaction (ND) across all replicates under the assay conditions employed (Table 3). Although the ammonia vapor assay is widely used as a rapid screening method, it should be regarded as a presumptive approach and does not constitute direct confirmation of mycotoxin production (Saito and Machida 1999). Furthermore, while this assay targets aflatoxin‐associated pathways typically found in other *Aspergillus* sections, black aspergilli are known potential producers of other specific mycotoxins, such as ochratoxin A and fumonisin B2. Since analytical chromatographic quantification (such as HPLC‐MS) was outside the scope of this primary genomic characterization framework, the safety profile of these isolates remains preliminary. Detailed chromatographic validation will be mandatory in future investigations to firmly exclude the presence of ochratoxin A and fumonisin B2 before any industrial scale‐up.

**Table 3:** Qualitative assessment of aflatoxin‐associated reactions in *Aspergillus* isolates grown on Coconut Extract Agar (CEA) and evaluated using the ammonia vapor assay.

Isolate codes	Fungal species	Ammonia vapor assay
E1	*Aspergillus* sp_1_.	ND
A2	*Aspergillus candidus*	++++
G3	*Aspergillus fumigatus*	ND
E2	*Aspergillus* sp_2_.	ND
O5	*Aspergillus flavus*	++++
E3	*Aspergillus* sp_3_.	ND
E4	*Aspergillus* sp_4_.	ND

*Note:* Isolates were cultivated on Coconut Extract Agar (CEA) and screened for aflatoxin‐associated reactions using the ammonia vapor assay. Reactions were evaluated qualitatively according to colony color changes after ammonia exposure. ND: no detectable reaction; +: weak reaction; ++: moderate reaction; +++: strong reaction; ++++: very strong reaction.

Regarding the acidification screening on modified Czapek‐Dox agar, quantitative measurements of the yellow halo diameters revealed significant variations among the four selected isolates (*p* < 0.05). Isolate E2 exhibited the largest acidification halo, reaching a mean diameter of 57.50 ± 1.15 mm. Isolates E3 and E1 showed intermediate activities with mean diameters of 44.50 ± 0.70 and 41.40 ± 0.82 mm, respectively (Table 4). Isolate E4 displayed the lowest acidification capacity, with a mean halo diameter of 28.50 ± 0.60 mm. These quantitative results demonstrate significant differences in acidification capacity among the four environmental isolates, with E2 exhibiting the strongest acidification phenotype under the assay conditions. Because halo formation reflects medium acidification rather than the specific production of citric acid, these results were interpreted as a preliminary screening criterion and were subsequently complemented by quantitative citric acid determination during solid‐state fermentation. Similar screening approaches based on halo boundaries have been widely employed for the preliminary selection of citric acid‐producing fungi (Iqbal et al. 2015).

**Table 4:** Quantitative assessment of acidification halo diameters and selection of candidate *Aspergillus* isolates for genomic analysis.

Isolate code	Preliminary identification	Acidification potential	Yellow halo diameter (mm)	Selection for genomic analysis
E1	*Aspergillus* sp. 1	Very strong	41.40 ± 0.82^b^	Yes
A2	*Aspergillus candidus*	Moderate	20.10 ± 0.50^d^	No
G3	*Aspergillus fumigatus*	Not detected	0.00 ± 0.00	No
E2	*Aspergillus* sp. 2	Very strong	57.50 ± 1.15^a^	Yes
O5	*Aspergillus flavus*	Not detected	0.00 ± 0.00	No
E3	*Aspergillus* sp. 3	Very strong	44.50 ± 0.70^b^	Yes
E4	*Aspergillus* sp. 4	Very strong	28.50 ± 0.60^c^	Yes

*Note:* Values represent the mean of three independent experimental replicates ± standard deviation (*n* = 3). Non‐producing isolates (G3 and O5) were excluded from the statistical model due to an absolute absence of variance. For the producing strains, different superscript letters (*a, b, c, d*) within the column indicate statistically significant differences according to Tukey's Honestly Significant Difference (HSD) post‐hoc test (*p* < 0.001).

Interestingly, the isolates showing the highest acidification activities corresponded to those lacking detectable aflatoxin‐associated reactions. Although no causal relationship can be established from the present data, this observation supports their prioritization as candidates for further investigation.

### 3.3 Taxonomic Characterization of Selected Isolates

Taxonomic profiling revealed clear differences among the four selected isolates. At the higher taxonomic levels, isolates E1, E2, and E3 displayed highly similar profiles, characterized by the predominance of Ascomycota, Eurotiomycetes, Eurotiales, and *Aspergillus* at the phylum, class, order, and genus levels, respectively (Figures 2, 3, 4, 5). These patterns were consistent with their morphological characteristics and supported their affiliation with black aspergilli belonging to Aspergillus section Nigri (Samson et al. 2014; Houbraken et al. 2020).

**Figure 2:**
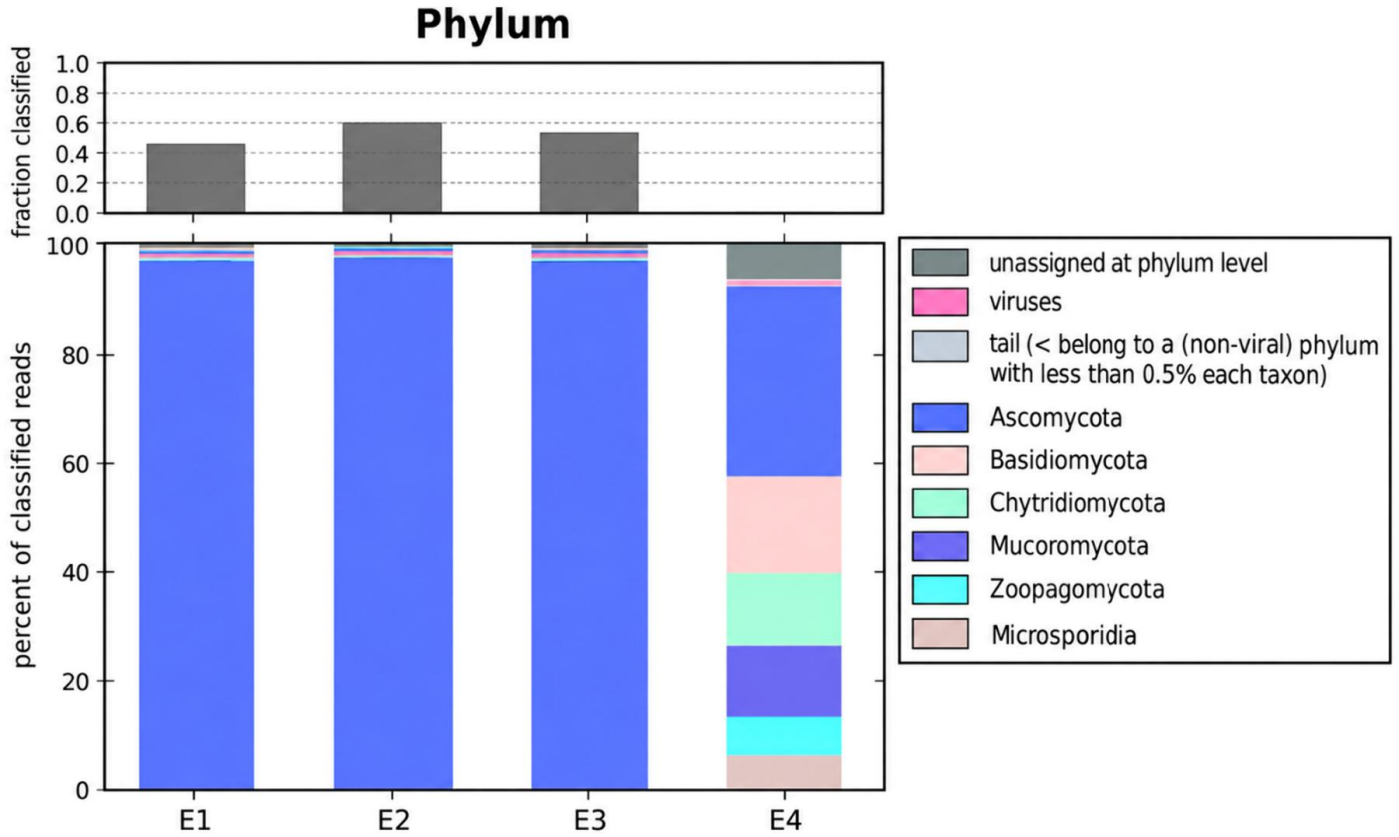
Phylum‐level taxonomic composition of sequenced environmental fungal isolates. Relative distribution of fungal phyla identified by Kaiju taxonomic profiling of Illumina sequencing data from isolates E1‐E4. E1‐E3 were predominantly assigned to Ascomycota, whereas E4 showed a more heterogeneous taxonomic profile.

**Figure 3:**
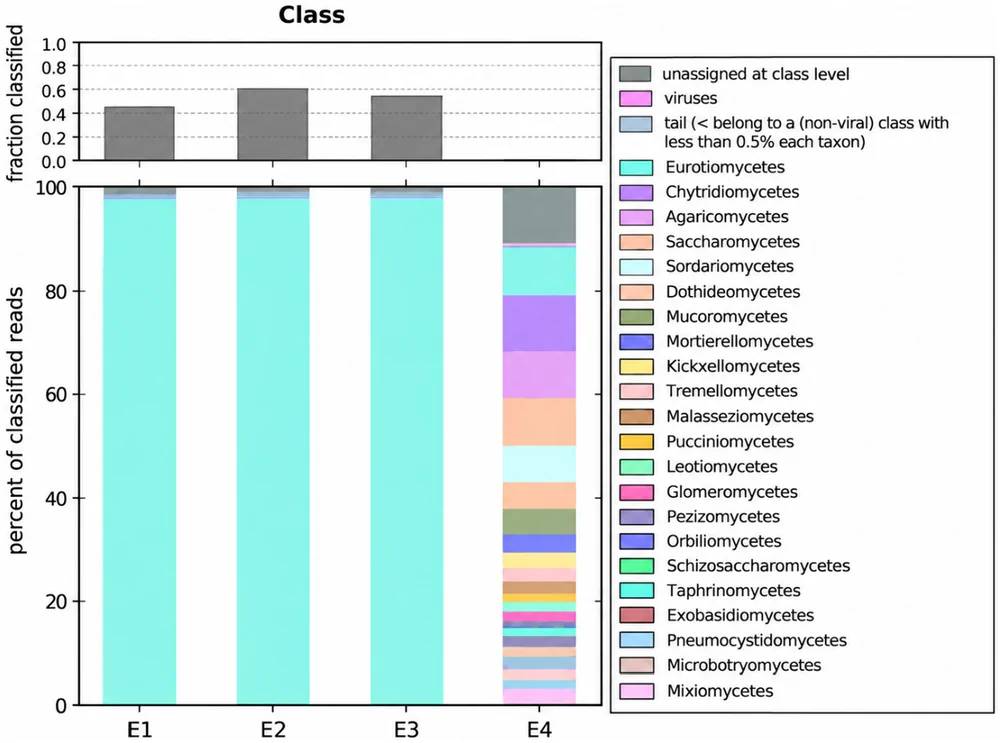
Class‐level taxonomic composition of sequenced environmental fungal isolates. Relative distribution of fungal classes identified by Kaiju taxonomic profiling of isolates E1‐E4. Eurotiomycetes predominated in E1‐E3, whereas E4 showed a more heterogeneous distribution of taxonomic assignments.

**Figure 4:**
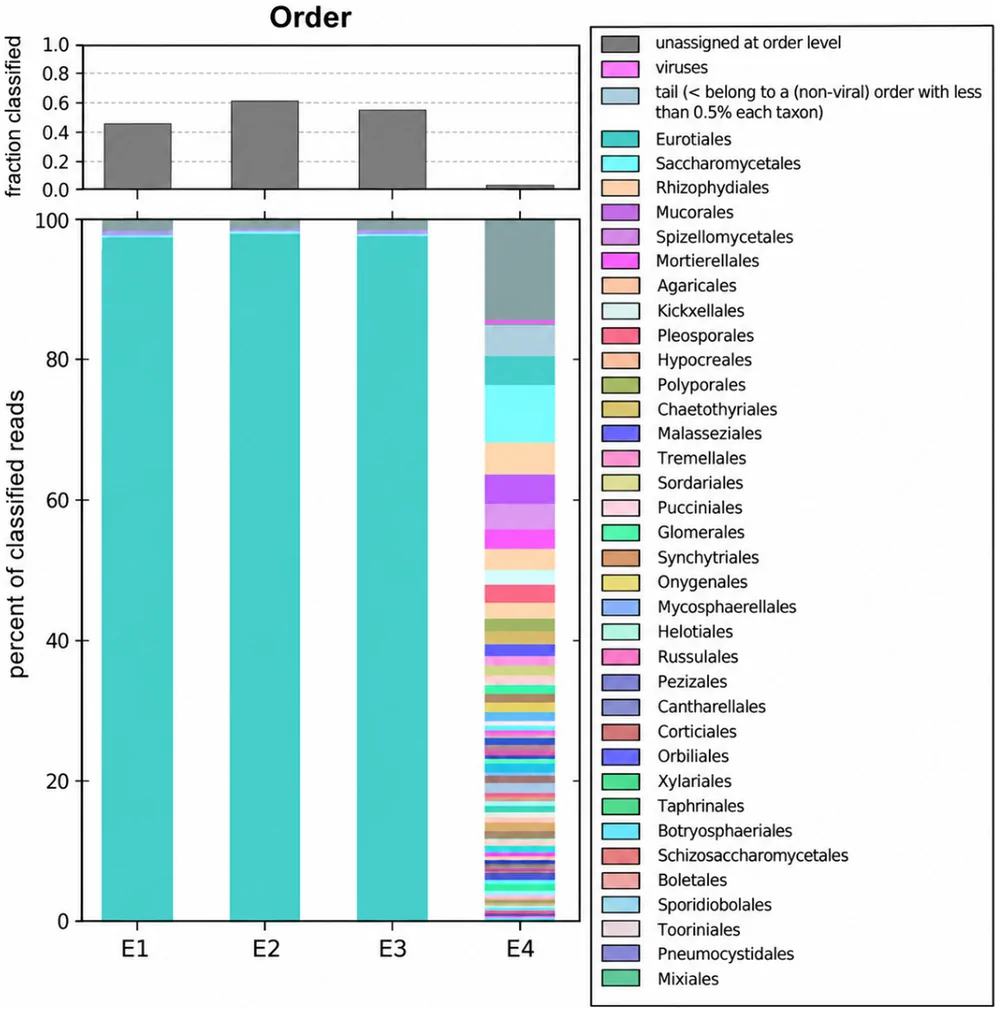
Order‐level taxonomic composition of sequenced environmental fungal isolates. Relative distribution of fungal orders identified by Kaiju taxonomic profiling of isolates E1‐E4. Eurotiales predominated in E1‐E3, whereas E4 showed a more heterogeneous distribution of taxonomic assignments.

**Figure 5:**
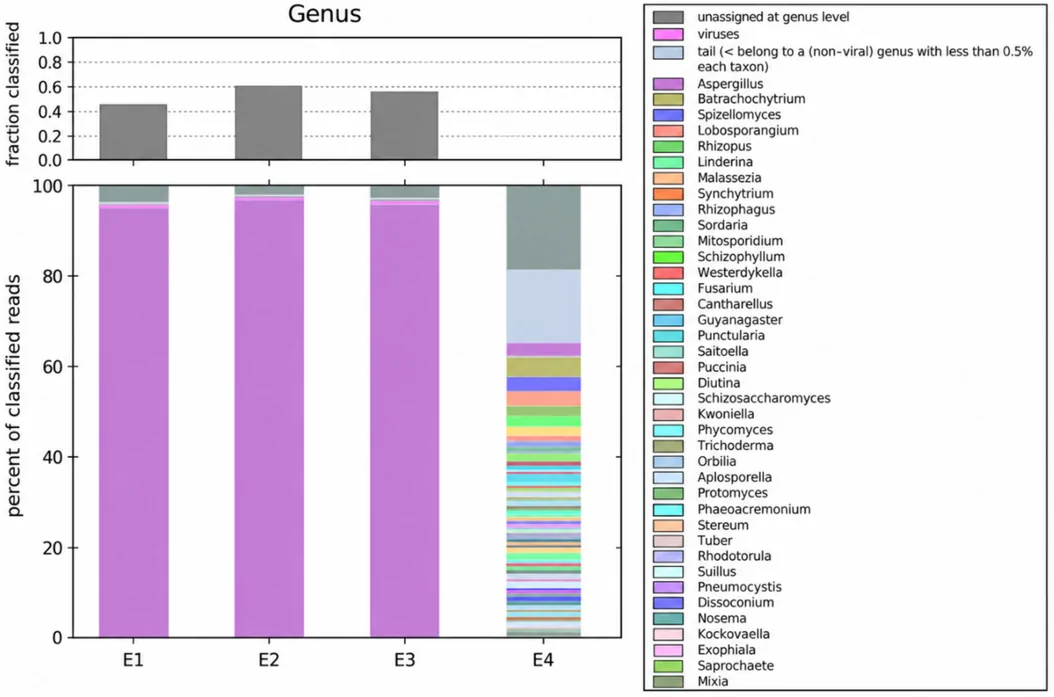
Genus‐level taxonomic composition of sequenced environmental fungal isolates. Relative distribution of fungal genera identified by Kaiju taxonomic profiling of isolates E1‐E4. *Aspergillus* predominated in E1‐E3, whereas E4 showed a more heterogeneous taxonomic profile.

The genomic assemblies of E1, E2, and E3 also showed sizes compatible with those generally reported for members of the *A. niger* species complex. Assembly sizes ranged from 29.95 to 35.76 Mb, while isolate E2 displayed the highest assembly continuity, with a scaffold N50 of 8211 bp, together with the highest BUSCO completeness value of 88.2% among the isolates evaluated (Table 5). In contrast, E4 exhibited a markedly reduced assembly size of 4.56 Mb and an atypical genomic profile. This unusually restricted assembly, together with the difficulty in performing standardized fungal lineage benchmarking for E4, indicates that its genomic dataset should be interpreted cautiously and does not support a confident species‐level assignment. The importance of genome completeness and assembly quality for reliable taxonomic and functional interpretation of Aspergillus genomes has previously been emphasized (Ellena and Steiger 2022).

**Table 5:** Genome assembly and sequencing quality metrics of the environmental *Aspergillus* isolates.

Isolates	Genome Size (Mb)	Scaffold N50 (bp)	GC content (%)	BUSCO completeness (%)	Sequencing depth (×)	Genome coverage (%)
E1	31.54	2520	50.16	39.8	3.5	> 95
E2	35.76	8211	50.18	88.2	7.5	> 95
E3	32.53	2109	50.23	34.6	3.5	> 95
E4	4.56	22,409	50.94	*n.a	8.5	> 95

*Note:* Assembly statistics were generated using SPAdes and QUAST from Illumina paired‐end sequencing data. Genome completeness was assessed using BUSCO v5.8.0 against the Eurotiales_odb10 lineage dataset. n.a., not available; BUSCO completeness was not determined for E4.

At the species‐reference level, reads assigned to *A. niger* represented 64%, 92%, and 65% of the classified reads for isolates E1, E2, and E3, respectively (Table 6). Additional assignments to closely related species, including *A. welwitschiae* and *A. luchuensis*, were also observed, particularly for E1 and E3. Such mixed assignments are not unexpected within *Aspergillus* section *Nigri*, where several closely related species share a high degree of sequence similarity and can be difficult to discriminate using reference‐based classification or individual molecular markers (Perrone et al. 2011; Samson et al. 2014; da Silva et al. 2020). The higher proportion of reads assigned to *A. niger* in E2 therefore indicates a stronger genome‐wide similarity to available *A. niger* reference sequences under the classification approach used, but should not be interpreted as definitive evidence that E2 represents *A. niger* sensu stricto. Similarly, the mixed assignments observed for E1 and E3 do not establish hybrid status or definitive affiliation with any individual species.

**Table 6:** Species‐level taxonomic affiliation of sequenced isolates within the Aspergillus niger species complex.

Isolates	Assigned species	Read count	Total BP mapped	Total BP mismatch	Relative abundance (%)
E1	*Aspergillus niger*	234,257	14,299,679	152,290	64
	*Aspergillus welwitschiae*	79,941	4,919,290	52,869	27
	*Aspergillus luchuensis*	11,071	913,606	11,715	8
E2	*Aspergillus niger*	555,727	38,835,378	177,450	92
	*Aspergillus welwitschiae*	31,227	2,098,220	26,824	6
	*Aspergillus luchuensis*	3792	343,358	6612	1
E3	*Aspergillus niger*	127,390	8,508,576	102,558	65
	*Aspergillus welwitschiae*	43,448	2,914,951	35,453	27
	*Aspergillus luchuensis*	4881	476,256	6883	7
E4	Uncategorized	—	—	—	—

*Note:* Taxonomic affiliation was performed by mapping sequencing reads against reference genomes belonging to the *Aspergillus niger* species complex. Relative abundance represents the proportion of classified reads assigned to each reference species. Isolates E1, E2, and E3 showed predominant read assignments to *A. niger* reference genomes, whereas E4 could not be confidently assigned to any of the reference species evaluated and was therefore retained as taxonomically unresolved.

Read count: Number of DNA fragments aligned to the genome; Total BP mapped: Total number of correctly aligned base pairs; Total BP mismatch: Number of mismatched base pairs during alignment.

Considering the convergence of morphological observations, higher‐level taxonomic profiles (Figures 2, 3, 4, 5), species‐reference assignments (Table 6), and genome assembly characteristics (Table 5), isolates E1, E2, and E3 were therefore conservatively regarded as members of the *Aspergillus niger* species complex. This terminology avoids overinterpretation of the reference‐based genomic assignments while remaining consistent with the available phenotypic and genomic evidence. Robust discrimination among closely related members of section *Nigri* would require targeted molecular characterization combined with dedicated multilocus phylogenetic analysis using discriminatory loci such as β‐tubulin (benA) and calmodulin (CaM).

In contrast, E4 displayed a markedly different genomic and taxonomic profile. Its assembly size was 4.56 Mb, and the available sequence data did not support confident assignment to the *A. niger* species complex or to another specific *Aspergillus* species. E4 was therefore retained as taxonomically unresolved. Further targeted molecular and phylogenetic analyses will be required to establish its taxonomic identity.

From a biotechnological perspective, the affiliation of E1‐E3 with the *A. niger* species complex is relevant because *A. niger* and related black aspergilli are widely studied for organic acid production, particularly citric acid (Karaffa and Kubicek 2003; Cairns et al. 2018; Latif et al. 2025). Nevertheless, taxonomic affiliation alone does not predict citric acid production performance. The taxonomic findings were therefore interpreted together with the quantitative acidification phenotype, functional genomic annotations, and solid‐state fermentation results presented in the subsequent sections.

### 3.4 Comparative Genomic Relationships Among Isolates

Comparative analysis based on shared genomic features revealed substantial similarities among isolates E1, E2, and E3 (Figure 6). These isolates shared a large proportion of the genomic features considered in the analysis, consistent with the taxonomic profiles supporting their affiliation with the *Aspergillus niger* species complex. In contrast, E4 displayed a lower degree of similarity with E1‐E3, in agreement with its atypical and taxonomically unresolved profile.

**Figure 6:**
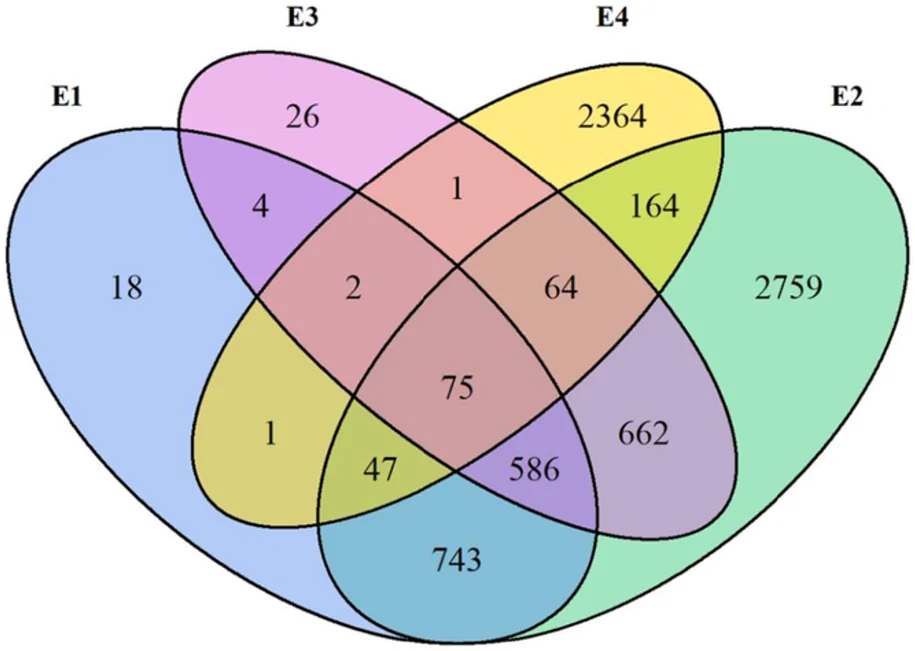
Distribution of shared and isolate‐specific annotated genomic features among isolates E1‐E4. Venn diagram showing the distribution of annotated genomic features shared among or specific to isolates E1‐E4. Numbers indicate the number of features associated with each isolate or combination of isolates.

Hierarchical clustering based on annotated gene content further illustrated these relationships (Figure 7). E1 and E3 clustered at the shortest genomic distance, whereas E2 formed a closely related branch. E4 remained separated from the E1‐E3 group. These results indicate greater similarity in annotated gene content among E1‐E3 than between these isolates and E4. However, because the clustering analysis was based on genome‐derived gene content rather than on a dedicated phylogenetic framework, the observed relationships should not be interpreted as evidence of species‐level evolutionary relationships.

**Figure 7:**
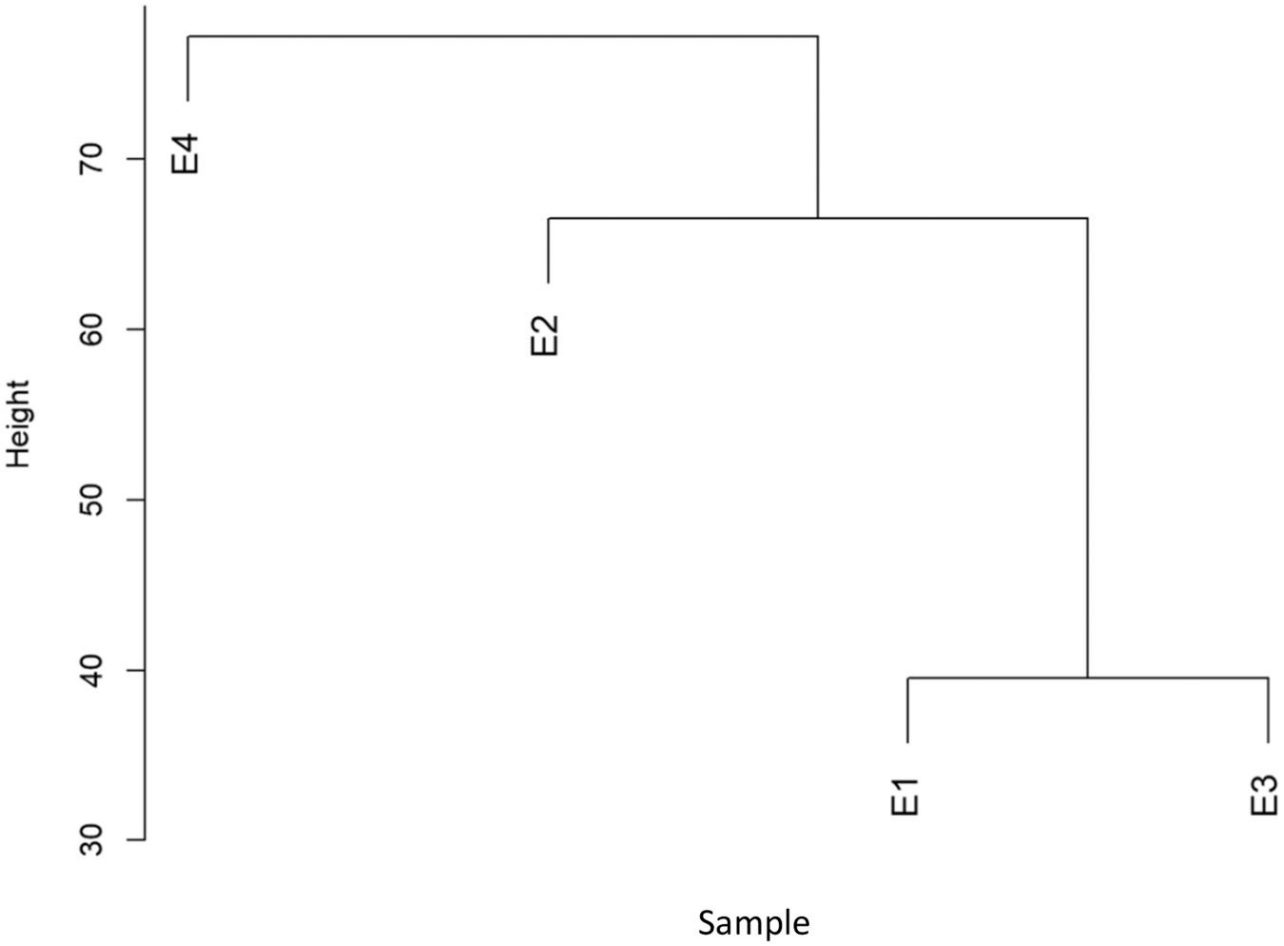
Hierarchical clustering of sequenced isolates based on annotated gene‐content similarity. Dendrogram illustrating similarities among isolates E1‐E4 based on genome‐derived annotated gene content. E1 and E3 clustered at the shortest distance, followed by E2, whereas E4 formed a separate branch. The clustering represents descriptive genomic similarity and should not be interpreted as a phylogenetic relationship or definitive taxonomic classification.

The similarity observed among E1‐E3 is consistent with the genomic diversity previously reported among closely related black aspergilli, in which isolates may share substantial genomic content while retaining strain‐ or lineage‐specific differences (Andersen et al. 2011; Vesth et al. 2018). Accordingly, the comparative genomic patterns observed here provide complementary support for the grouping of E1‐E3 within the *A. niger* species complex but do not replace multilocus or genome‐scale phylogenetic analyses for definitive taxonomic delimitation.

### 3.5 Functional Potential Associated With Citrate Metabolism

Functional annotation identified predicted functions associated with several key enzymes involved in citrate metabolism, including citrate synthase (EC 2.3.3.1), aconitase (EC 4.2.1.3), isocitrate dehydrogenase (EC 1.1.1.41/42), and isocitrate lyase (EC 4.1.3.1) (Figure 8). These enzymes participate in central carbon metabolism and are associated with citrate formation and utilization through the tricarboxylic acid cycle and related pathways. The presence of these predicted functions across the selected isolates indicates that their genomes contain the principal metabolic components associated with citrate metabolism.

**Figure 8:**
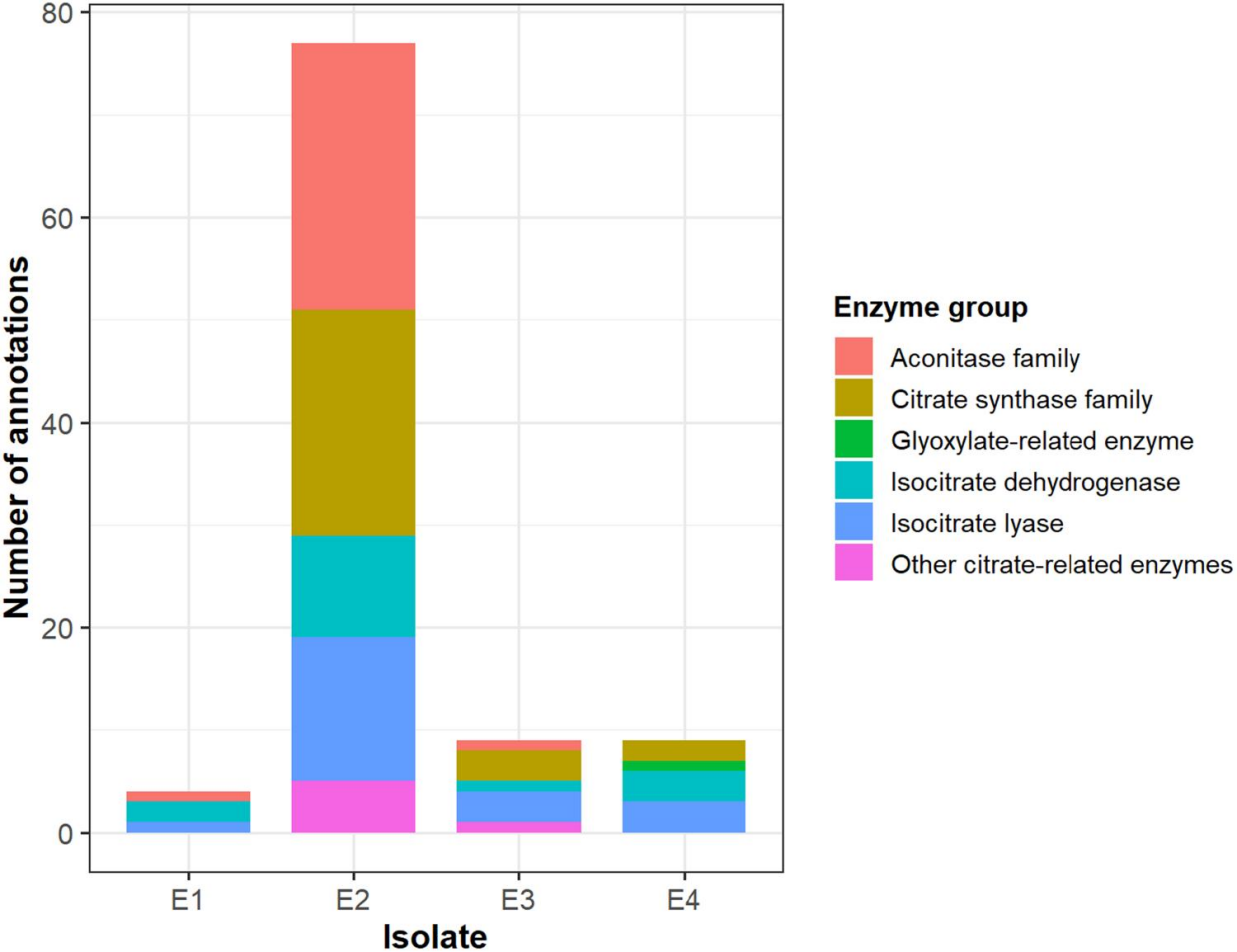
Distribution of predicted functions associated with citrate metabolism in sequenced *Aspergillus* isolates. Predicted functions associated with citrate metabolism were identified through EggNOG‐mapper functional annotation of isolates E1–E4 and grouped into major functional categories, including citrate synthase, aconitase, isocitrate dehydrogenase, isocitrate lyase, glyoxylate‐related enzymes, and other citrate‐associated functions. The number of annotations represents the occurrence of predicted functions assigned to each category. E2 showed the highest representation of predicted citrate metabolism‐associated functions. These genome‐derived annotations represent predicted metabolic potential and do not demonstrate gene expression or enzyme activity.

Among the isolates, E2 showed the highest representation of predicted functions associated with citrate metabolism (Figure 8). This genomic pattern is of particular interest because citrate synthase catalyzes the condensation of acetyl‐CoA and oxaloacetate to form citrate, whereas aconitase and isocitrate dehydrogenase participate in subsequent citrate utilization within the tricarboxylic acid cycle. Citric acid accumulation in *A. niger*

is nevertheless a complex physiological process controlled not simply by the presence of individual genes but by carbon flux, enzyme regulation, substrate availability, trace elements, pH, and other cultivation conditions (Karaffa and Kubicek 2003; Papagianni 2007).

### 3.6 Functional Potential for Carbohydrate Degradation

Functional annotation revealed predicted functions associated with carbohydrate degradation, including α‐amylases, β‐glucosidases, glucanases, and glycosyl hydrolases (Supporting Information S1: Figure S1). These enzymatic functions are involved in the hydrolysis of complex carbohydrates into simpler sugars that can subsequently enter central carbon metabolism. Their presence is therefore relevant to the potential utilization of carbohydrate‐rich substrates by the selected isolates.

Among the isolates, E2 showed the highest representation of predicted functions associated with carbohydrate degradation (Supporting Information S1: Figure S1), whereas E1 and E3 displayed intermediate profiles. These differences suggest variation in the predicted capacity of the isolates to mobilize complex carbon sources. Such metabolic versatility may be relevant for citric acid production from agro‐industrial substrates, where efficient substrate hydrolysis and carbon assimilation can influence the availability of fermentable sugars for central metabolism (Papagianni 2007; Behera 2020).

However, the presence and relative representation of these predicted functions should not be interpreted as direct evidence of enzyme activity or substrate‐conversion efficiency. Genome‐derived functional annotations indicate metabolic potential, whereas the actual utilization of a substrate depends on gene expression, enzyme secretion and activity, substrate composition, and cultivation conditions.

Taken together, the taxonomic profile, acidification phenotype, and functional annotations identified E2 as the most promising candidate among the isolates investigated. Nevertheless, genomic annotation alone cannot establish whether these predicted metabolic features translate into enhanced citric acid production. The quantitative solid‐state fermentation experiments presented in Section 3.7 were therefore used to determine whether the predicted genomic pattern was associated with higher measured citric acid production under the experimental conditions tested.

### 3.7 Quantitative Citric Acid Production During Solid‐State Fermentation

To complement the preliminary acidification screening and determine whether the selected isolates differed in their capacity to produce citric acid under fermentation conditions, quantitative citric acid production was monitored during solid‐state fermentation. Significant differences were observed among the isolates (one‐way ANOVA, *p* < 0.001), confirming substantial variation in their production profiles under the experimental conditions tested (Supporting Information S1: Figure S2).

E2 consistently exhibited the highest measured citric acid concentration during fermentation and reached a maximum value of 0.302 g/kg at day 12. Under the same experimental conditions, the maximum values recorded for E1, E3, and E4 were 0.240, 0.180, and 0.140 g/kg, respectively. The maximum value obtained for E2 was significantly higher than those of the other isolates (Tukey's HSD, *p* < 0.001). These quantitative results were consistent with the preliminary acidification assay, in which E2 also exhibited the largest acidification halo.

Citric acid production increased progressively during fermentation before tending toward a plateau between days 12 and 15. Such a production pattern may reflect changes in nutrient availability and fungal metabolic activity during fermentation. However, because residual sugars, pH evolution, biomass formation, and other fermentation parameters were not monitored simultaneously, the physiological mechanisms underlying this production profile cannot be determined from the present experiment. The observed plateau should therefore be interpreted descriptively rather than attributed to a specific metabolic limitation.

Although E2 exhibited the highest measured citric acid concentration among the isolates tested, the concentrations obtained in the present study (0.140–0.302 g/kg) remain substantially lower than those reported for optimized industrial citric acid fermentation systems. This difference is not unexpected because the present experiments were designed primarily to compare environmental isolates under a common set of experimental conditions rather than to optimize citric acid production. Industrial production by A. niger depends on multiple parameters, including carbon source concentration, nitrogen limitation, trace metal availability, pH, oxygen transfer, inoculum characteristics, and fermentation configuration (Karaffa and Kubicek 2003; Papagianni 2007). Consequently, the present results should be considered a basis for isolate selection and subsequent process optimization rather than an indication of industrial production performance.

Interestingly, the production hierarchy observed during fermentation (E2 > E1 > E3 > E4) paralleled the relative pattern observed in the genome‐derived functional annotations. E2, which exhibited the highest measured citric acid concentration, also showed the greatest representation of predicted functions associated with citrate metabolism and carbohydrate degradation (Figure 8 and Supporting Information S1: Figure S1). This concordance represents an association between genomic predictions and the observed fermentation phenotype but does not establish a causal relationship. Demonstrating such a relationship would require additional evidence from gene‐expression, enzyme‐activity, proteomic, or metabolic‐flux analyses.

Finally, although the calibrated colorimetric assay provided quantitative estimates of citric acid production and enabled comparison among isolates, it does not provide the same analytical specificity as chromatographic methods. The measured concentrations should therefore be interpreted as quantitative supporting evidence for citric acid production under the tested conditions. Confirmation using HPLC or another validated chromatographic method would strengthen compound‐specific identification and provide a more robust basis for subsequent fermentation‐process optimization.

### 3.8 Study Limitations

Several limitations should be considered when interpreting the findings of this study. First, although citric acid production was quantitatively evaluated during solid‐state fermentation using a colorimetric assay calibrated with a commercial citric acid standard, chromatographic confirmation by HPLC or an equivalent highly specific analytical method was not performed. The colorimetric method therefore provides quantitative supporting evidence for citric acid production but does not offer the same compound‐specific analytical confirmation as chromatographic methods. Future studies should include HPLC‐based confirmation of citric acid identity and concentration, together with optimization of fermentation parameters and determination of productivity and substrate conversion yields under controlled conditions.

Second, the ammonia vapor assay was used as a rapid presumptive screening method for aflatoxin‐associated reactions. Although no detectable aflatoxin‐associated reaction was observed for E1–E4 under the assay conditions, this result should not be interpreted as demonstrating the complete mycotoxin safety of the isolates. Other potentially relevant mycotoxins, including ochratoxin A and fumonisin B2, were not directly quantified. Comprehensive safety assessment using validated chromatographic and/or molecular approaches will therefore be required before any potential food‐related or industrial application (Frisvad et al. 2011; Silva et al. 2020).

Third, taxonomic characterization of E1‐E3 was based on the convergence of morphological observations and whole‐genome taxonomic profiling. Although these data supported their conservative affiliation with the *A. niger* species complex, they do not provide definitive species‐level identification within *Aspergillus* section *Nigri*. E4 remained taxonomically unresolved based on the available evidence. Targeted molecular characterization combined with dedicated multilocus phylogenetic analysis using discriminatory markers such as β‐tubulin (benA) and calmodulin (CaM) will therefore be required for robust species‐level delimitation (Samson et al. 2014).

Finally, functional analyses were based on genome‐derived annotations and therefore represent predicted metabolic potential rather than experimentally demonstrated gene expression, enzyme activity, or metabolic flux. Although the relative pattern of predicted citrate metabolism‐ and carbohydrate degradation‐associated functions was consistent with the quantitative fermentation phenotype, particularly for E2, this concordance does not establish a causal relationship. Integration of transcriptomic, proteomic, enzymatic, and metabolic‐flux analyses would be required to determine the functional contribution of individual genes and pathways to citric acid production.

## 4 Conclusion

This study combined conventional microbiological screening, quantitative phenotypic assessment, solid‐state fermentation, and genome‐informed analyses to characterize environmental *Aspergillus* isolates with potential relevance for citric acid production. Among the 24 *Aspergillus* isolates recovered from environmental samples, four isolates (E1‐E4) were selected based on their acidification capacity and the absence of detectable aflatoxin‐associated reactions under the conditions of the ammonia vapor assay. Morphological characterization and whole‐genome taxonomic profiling supported the affiliation of E1, E2, and E3 with the *Aspergillus niger* species complex, whereas E4 remained taxonomically unresolved. The ITS region and the β‐tubulin (benA) locus were successfully retrieved in silico from the genomic assemblies of E1‐E3, providing complementary taxonomic sequence information. However, definitive species‐level identification would require dedicated multilocus phylogenetic analysis. Quantitative acidification measurements and solid‐state fermentation revealed significant differences among the isolates, with E2 exhibiting the strongest acidification phenotype and the highest measured citric acid concentration under the experimental conditions tested. Functional annotation also showed that E2 had the highest representation of predicted functions associated with citrate metabolism and carbohydrate degradation. However, these genome‐derived annotations represent predicted metabolic potential and do not demonstrate gene expression, enzyme activity, or metabolic flux. Overall, the convergence of quantitative phenotypic data, fermentation results, and genome‐informed analyses identifies E2 as the most promising candidate among the isolates evaluated for further citric acid production studies. Nevertheless, chromatographic confirmation of citric acid, fermentation‐process optimization, comprehensive mycotoxin assessment, and multilocus taxonomic validation will be required before any potential industrial application is considered. This study demonstrates the value of integrating quantitative phenotypic screening with genome‐informed analyses for the characterization and prioritization of environmental fungal isolates of biotechnological interest.

## Author Contributions

**Wimbélé Epiphanie Kouman:** conceptualization, investigation, methodology, formal analysis; data curation, writing – original draft. **Okran Beyosse Christophe Kacou:** data curation, formal analysis, visualization, validation, writing – original draft, investigation. **Sansan Kambou:** conceptualization, writing – review and editing, supervision, methodology. **Ousmane Fofana:** investigation, methodology, formal analysis, data curation. **Djédoux Maxime Angaman:** conceptualization, writing – review and editing, methodology, formal analysis, data curation, supervision, validation.

## Funding

The authors have nothing to report.

## Ethics Statement

The authors have nothing to report.

## Conflicts of Interest

The authors declare no conflicts of interest.

## Supporting information


**Figure S1:** Comparative distribution of carbohydrate‐active enzyme families in environmental *Aspergillus* isolates. Relative abundance of predicted carbohydrate‐active enzyme families identified through EggNOG functional annotation of isolates E1‐E4. Enzyme groups include α‐amylases, α‐glucosidases, glucan‐degrading enzymes, glycosyl hydrolases, and other carbohydrate‐related proteins.
**Figure S2:** Time‐course of citric acid production by selected *Aspergillus* isolates during solid‐state fermentation. Citric acid production by isolates E1‐E4 was monitored over 15 days using industrial pineapple waste (DIAaa) as substrate. Values are expressed as g/kg of substrate and represent mean ± SD of three independent experimental replicates (*n* = 3). Overall differences among isolates were assessed by one‐way ANOVA (*p* < 0.001), followed by Tukey's HSD test. Different lowercase letters indicate significant differences among isolates (*p* < 0.001).

## Data Availability

The whole‐genome sequencing datasets generated in this study are being deposited in the NCBI Sequence Read Archive (SRA) under BioProject accession PRJNA1526301. The corresponding BioSample accession numbers are SAMN63036955, SAMN63036956, SAMN63036957, and SAMN63036958.
