# Supplementary material for: Integrated Phenotypic and Genome‐Informed Characterization of Environmental Aspergillus Isolates With Potential for Citric Acid Production
Source: Microbiologyopen. 2026 Nov 5;15(6):e70452. doi: 10.1002/mbo3.70452 (PMC13740965; doi:10.1002/mbo3.70452)
Supplement: Supplementary file 1 [file MBO3-15-e70452-s001.docx]

**Supplementary Figure S1. Comparative distribution of carbohydrate-active enzyme families in environmental *Aspergillus* isolates**

Relative abundance of predicted carbohydrate-active enzyme families identified through EggNOG functional annotation of isolates E1-E4. Enzyme groups include α-amylases, α-glucosidases, glucan-degrading enzymes, glycosyl hydrolases, and other carbohydrate-related proteins.

**Supplementary Figure S2.** Time-course of citric acid production by selected *Aspergillus* isolates during solid-state fermentation.

Citric acid production by isolates E1–E4 was monitored over 15 days using industrial pineapple waste (DIAaa) as substrate. Values are expressed as g/kg of substrate and represent mean ± SD of three independent experimental replicates (n = 3). Overall differences among isolates were assessed by one-way ANOVA (p < 0.001), followed by Tukey’s HSD test. Different lowercase letters indicate significant differences among isolates (p < 0.001).
